# Good vibrations: A review of vocal expressions of positive emotions

**DOI:** 10.3758/s13423-019-01701-x

**Published:** 2020-01-02

**Authors:** Roza G. Kamiloğlu, Agneta H. Fischer, Disa A. Sauter

**Affiliations:** Department of Psychology, University of Amsterdam, REC G, Nieuwe Achtergracht 129 B, PO Box 15900, 1001 NK Amsterdam, The Netherlands

**Keywords:** Vocal expression, Positive emotions, Acoustic features, Speech prosody, Nonverbal vocalizations

## Abstract

Researchers examining nonverbal communication of emotions are becoming increasingly interested in differentiations between different positive emotional states like interest, relief, and pride. But despite the importance of the voice in communicating emotion in general and positive emotion in particular, there is to date no systematic review of what characterizes vocal expressions of different positive emotions. Furthermore, integration and synthesis of current findings are lacking. In this review, we comprehensively review studies (*N* = 108) investigating acoustic features relating to specific positive emotions in speech prosody and nonverbal vocalizations. We find that happy voices are generally loud with considerable variability in loudness, have high and variable pitch, and are high in the first two formant frequencies. When specific positive emotions are directly compared with each other, pitch mean, loudness mean, and speech rate differ across positive emotions, with patterns mapping onto clusters of emotions, so-called emotion families. For instance, pitch is higher for epistemological emotions (amusement, interest, relief), moderate for savouring emotions (contentment and pleasure), and lower for a prosocial emotion (admiration). Some, but not all, of the differences in acoustic patterns also map on to differences in arousal levels. We end by pointing to limitations in extant work and making concrete proposals for future research on positive emotions in the voice.

When interacting with others, we rely on different communication channels, including nonverbal expressions in the face, voice, and body. The voice constitutes a particularly important means of communication. Vocal signals have been shown to convey not only relatively enduring features like age and gender, but also a wide range of transitory states such as health and power (Kreiman & Sidtis, 2011). It has been proposed that the human voice also conveys emotional states, each characterized by a unique acoustic profile (e.g., Banse & Scherer, 1996; Scherer, Banse, Wallbott, & Goldbeck, 1991). A number of studies support the idea of emotion-specific patterns of acoustic features for discrete negative emotions, in that acoustic profiles of several negative emotions, including anger, fear, and sadness, have been reported to show considerable differentiation (e.g., Banse & Scherer, 1996; Juslin & Laukka, 2001; van Bezooijen, 1984; Pollermann & Archinard, 2002). To date, attempts to acoustically differentiate between vocal expressions of different emotions, however, have been primarily focused on negative emotions. Most research has included a very limited number of positive compared to negative emotions (Sauter & Scott, 2007) or has used a single positive emotion, happiness, as an umbrella term. This makes it challenging to establish whether there is differentiation between vocal expressions of positive emotions. Even though research on vocalizations of positive emotions is scarce compared to negative emotions, different positive emotions have been suggested to be characterized by distinct patterns of cognition, physiological responding, and behaviour, including nonverbal expressions (Shiota et al., 2014; Shiota et al., 2017).

## A functional approach to differentiation of positive emotions

Many contemporary emotion theorists agree with the suggestion that a host of discrete negative emotions serve distinct adaptive purposes relating to different types of threats and challenges (e.g., Adolphs & Andler, 2018; Cosmides & Tooby, 2000; Ekman, 1992; Shiota et al., 2014; Tooby & Cosmides, 2008). Positive emotions are also considered important to human survival, because they coordinate cognitive, physiological, and behavioural mechanisms and facilitate adaptive responses to opportunities, such as affiliation and cooperation (Shiota et al., 2014). Biopsychosocial environments encountered in daily life might elicit a variety of positive emotions, with different positive emotions serving different adaptive purposes. Discrete positive emotions have thus been suggested to have evolved to facilitate fitness-enhancing responses to different kinds of evolutionarily recurring opportunities (e.g., Cosmides & Tooby, 2000; Keltner, Haidt, & Shiota, 2006). For instance, finishing first in an important competition might elicit different fitness-enhancing responses than would watching a beautiful vista from a mountaintop.

Functional approaches take a prototypical event that elicits a specific positive emotion (e.g., amusement, awe, pride, tenderness) as a starting point, and attempt to explain the overall adaptive function of the emotion to that kind of event (Cosmides & Tooby, 2000). Given that discrete positive emotions serve adaptive functions that are suited to different types of kinds of opportunities, it follows that they may involve different expressive signals (Shiota et al., 2017), such as distinct acoustic patterns in the voice. This raises the question of whether discrete positive emotions are expressed via vocal signals with different configurations of acoustic features.

Although emotions may serve different functions, they can share characteristics, thereby yielding higher-order groups of “families” of emotions (Ekman, 1992). Based on clustering of nonverbal expressions of positive emotions (facial and bodily expressions, speech prosody, and nonverbal vocalizations), researchers have proposed that positive emotions may cluster into emotion families of epistemological, savouring, prosocial, and agency-approach positive emotions (Sauter, 2017; Simon-Thomas, Keltner, Sauter, Sinicropi-Yao, & Abramson, 2009). Epistemological positive emotions refer to emotions involved in changes in individuals’ knowledge about the world and include amusement, interest, relief, and awe. Savouring positive emotions are triggered by thinking about or experiencing different kinds of sensory enjoyment and include contentment, sensory pleasure, and sexual desire. Prosocial positive emotions are linked to concern for others and include love, compassion, gratitude, and admiration. Agency approach positive emotions refer to emotions characterized by approach tendencies, and include elation and pride.

## Discrete positive emotions in the human voice

Humans produce a range of different nonverbal expressions in the voice: we laugh with amusement, sigh with relief, and cheer with triumph. In addition to nonverbal vocalizations, we might use words or sentences with different intonation patterns when we are in different positive emotional states. Indeed, the importance of distinguishing between different positive emotions in the domain of vocal signals has been noted by several theorists. In an early review of emotional vocalizations, Scherer (1986) emphasized the need to understand what the umbrella term “happiness” refers to in order to compare results from different research lines. More specifically, Ekman (1992) suggested that “happiness” be replaced by several discrete positive emotions. He hypothesized that a wider range of positive emotions may be conveyed by vocalizations than by facial expressions. However, it is only in recent years that empirical work has started to address the question of whether different positive emotions are associated with discrete vocal signatures. Increasingly, emotion researchers are starting to go beyond a single positive emotion and instead include vocal expressions of multiple positive emotions including achievement, amusement, contentment, pleasure, and relief (e.g., Anikin & Persson, 2016; Laukka et al., 2016; Lima, Castro, & Scott, 2013; Sauter & Scott, 2007).

It is worth noting that in previous literature, most studies have drawn inferences about the production of emotional expressions in the voice on the basis of the study of perception, particularly recognition accuracy (Sauter, 2017). There is empirical evidence showing that a number of distinct positive emotions can be accurately recognized from the voice (e.g., Sauter & Scott, 2007; Simon-Thomas et al., 2009), even across cultures and languages (e.g., Cordaro, Keltner, Tshering, Wangchuk, & Flynn, 2016; Laukka et al., 2013; Sauter, Eisner, Ekman, & Scott, 2010). Research on the recognition of emotions from vocal expressions thus demonstrates that human listeners can differentiate some positive emotions on the basis of vocal signals. Are there, then, any benefits of emotional vocal communication for the listener? One account of vocal communication proposes that vocalizations of emotions provide information that is to the advantage of both the producer and the receiver. On this view, vocal communication transfers emotional information leading to different adaptive behavioural responses by receivers (Seyfarth et al., 2010). For instance, alarm calls produced by several species distinguish between predator types, and in response, receivers have developed different behavioural patterns (see Zuberbühler, 2009, for a review). According to this view, the transfer of information from producer to receiver, especially in close living social groups, is presumed to increase reproductive success for all. Another account of vocal communication argues that vocal communication of emotions has evolved to allow producers to affect the behaviours of receivers in a manner that is advantageous to the producer of the vocalizations, but not necessarily for the perceiver (Rendall, Owren, & Ryan, 2009). For example, humans use certain vocalizations to induce fear in order to control other animals (McConnell, 1991) or human infants (Fernald, 1992). Such vocalizations are explicitly intended to alter the behaviour of the receiver. Both of these views see vocal expressions as communicative. Within a communicative framework, vocalizations are referred to as signals. Another approach to vocalizations holds that vocalizations can provide information to others, even though the vocalization was not produced in order to communicate. In such a framework, vocalizations are considered cues (Wiley, 1983). It is, therefore, important to examine production of emotional vocalizations, that is, the patterns of expressive features in the voice that characterize specific emotions, as a crucial aspect of vocal communication.

## The current review

To date, reviews on vocal expression of emotions have focused primarily on negative emotions (Murray & Arnott, 1993; Scherer, 1986), or have examined broader topics such as comparing vocal expression and musical performance (Juslin & Laukka, 2003). However, in recent years, there is a rapidly growing body of evidence on vocal expressions of positive emotions. The present paper provides a review of the acoustic profiles of vocalizations of all positive emotions that have been studied to date. Specifically, we sought to examine whether there are distinct acoustic patterns associated with discrete positive emotions, and whether acoustic features can be grouped based on the functional similarity of positive emotions (emotion families). We also consider an alternative approach to defining emotional states, namely core affect dimensions: arousal (the degree of physiological alertness or attentiveness) and valence (the degree of pleasure or displeasure, positivity or negativity; Russell, 1980). Acoustic features of vocalizations are related to the producer’s affective state, which in turn relates to physiological changes including changes to vocal production machinery (Scherer, 1986). In particular, acoustic features of vocalizations might contain information about the producer’s arousal level (e.g., Filippi et al., 2017). For the purpose of the current review, we examine arousal, but not valence, since all positive emotions share positive valence. We thus consider explanations of acoustic variability of positive vocalizations based both on functional and arousal accounts.

By focusing on acoustic information, we aim to map discrete positive emotions onto physical features without relying on subjective measures such as self-report or listener judgments (although we include such information where available). First, we present an overview of the studies conducted to date, as well as a review of the terminology of positive emotions used in this literature. To be as comprehensive as possible, all studies including at least one positive emotion are included. Second, we specifically examine studies including either one positive emotion and a neutral baseline, or more than one positive emotion. We present a comparative review of these two groups of studies. We end by summarizing the available evidence, evaluating general design features of this body of empirical research, and making a number of recommendations for future research in this field.

Emotions in the voice can be expressed in several ways, including via semantics, speech prosody, and nonverbal vocalizations. Semantic information refers to the linguistic content of speech, such as for instance, the meaning of sentences such as ‘I am proud’ or ‘I am excited’. Linguistic meaning expressing emotions in language is complex and multifold (see Majid, 2012). The present review does not include studies on semantics of emotions. Rather, we focus on the acoustic features of vocalizations associated with positive emotions, as expressed via both speech prosody and nonverbal vocalizations. Speech prosody refers to the pattern of acoustic changes within verbal utterances, and is studied by examining speech (words, sentences) or pseudospeech (linguistically meaningless speech sounds) spoken in different emotional tones (see Juslin & Laukka, 2003). Nonverbal emotional vocalizations or affect bursts (Scherer, 1994), refer to nonspeech vocal sounds, such as laughs or screams.

A second constraint to our review is the emotional states that we examine: We include only studies investigating acoustic features of discrete positive emotions, such as joy, love, relief, pride, and amusement. Research on general positive affective states labelled only ‘general positive affect’ was excluded, as were studies examining only negative emotions. We thus included studies in which acoustic parameters of at least one positive emotion were investigated. Emotions were coded exactly as they were labelled by the authors. For example, if one study used the term *amusement* and the other *joy* for an emotion state, we would code these two studies as investigating amusement and joy, respectively, even if they were elicited by the same method.

In conducting this literature review, we reviewed research published in peer-reviewed journals using the databases PsychINFO, Google Scholar, and Web of Science. We also included reports listed in the computer science-oriented IEEE Xplore database, and unpublished doctoral dissertations available online. The following keywords were used separately and in combination: *voice, emotion, expression, acoustics, prosody, nonverbal.* We omitted nonempirical publications such as commentaries, reviews, and popular press articles. All English-language publications that reported empirical findings on acoustic features of vocalizations and that met the two criteria given above (i.e., a focus on speech prosody or nonverbal vocalizations and the inclusion of minimally one positive emotion), were included. The search was completed in January 2018 and yielded 108 studies.

### Overview of reviewed studies

Table 1 presents a summary of the 108 studies included in this review, reporting author(s), publication year, type of vocalization (speech prosody or nonverbal vocalizations), method used for eliciting vocalizations (acted, spontaneous, induced, or synthesized), emotion categories as labelled by the original authors, speaker information (gender and number of speakers and, where applicable, acting experience), and the acoustic features reported.

**Table 1:** Overview of research on acoustic parameters of positive emotions in the voice

Study No	Authors and year	Type	Method	Emotion Categories	Speakers	Acoustic measures
1	Abelin & Allwood (2000)	SP	A	**Joy,** anger, disgust, dominance, fear, sadness, shyness, surprise	1 male—nonprof. actor	*f*_o_, Int, SR
2	Al-Watban (1998)	SP	A	**Happiness,** anger, fear, sadness	8 male—prof. actors	*f*_o_, Int, SR
3	Anikin & Lima (2017)	NV	A, SN	**Amusement, pleasure,** anger, disgust, fear, pain, sadness	10 to 22 mixed male and female—taken from seven published corpora	*f*_o_, HNR, RMS, Energy, Interburst Interval, Duration, Spectral Slope, Voiced (%)
4	Anikin & Persson (2016)	NV	SN	**Amusement, joy, pleasure,** anger, disgust, effort, fear, pain, sadness	25 to 48 mixed male female or child—taken from YouTube videos	Energy, *f*_o_, HNR, Interburst Interval, Int
5	Aubergé et al. (2004)	SP	A, SN	**Confidence, joy, joy/surprise, positive concentration, positive surprise, satisfaction,** anxiety, anxiety/fear, deception/surprise, disgust, negative concentration, sadness, weariness, worried	“Some” prof. actors, “some” nonprof. actors	*f*_o_, SR
6	Aubergé & Cathiard (2003)	NV	A, SN	**Amusement**	3 prof. actors & 1 nonprof. actor	*f*_o_, Int, SR, Formants, Spectr.
7	Audibert et al. (2005)	SP	ST	**Happiness/joy, satisfaction,** anxiety, disappointment, disgust, resignation, sadness, worried	1 male prof. actor	*f*_o_, SR
8	Baldwin (1988)	SP	A	**Happiness,** anger, disgust, fear, sadness, surprise	6 male 6 female (3 prof. actors, 3 nonprof. actors for each gender)	Int., SR
9	Banse & Scherer (1996)	SP	A	**Elation, happiness, interest, pride,** anxiety, boredom, cold anger, contempt, despair, disgust, hot anger, panic, sadness, shame	6 male 6 female—prof. actors	*f*_o_, SR, Energy, Int, Spect,
10	Bänziger & Scherer (2005)	SP	A	**Calm joy, elated joy,** anxious fear, cold anger, depressed sadness, despaired sadness, hot anger, panic fear	4 male 5 female-prof. actors	*f*_o_ contour, Int
11	Banziger et al. (2013)	NV, SP	A	**Happiness/joy,** anger, fear, sadness	4 male 5 female & 5 male 5 female—prof. actor—taken from two corpora	*f*_o_, Int, SR, HNR
12	Baroni et al. (1997)	SP	A	**Happiness,** anger, sadness	3 singers and 3 prof. actors	*f*_o_, Int, SR
13	Baroni & Finarelli (1994)	SP	A	**Joyful,** aggressive, sad	3 singers and 3 prof. actors	Int, SR
14	Barrett & Paus (2002)	SP	I	**Happy,** sad	63 speakers—nonprof. actors	*f*_o_, Int, SR
15	Belin et al. (2008)	NV	A	**Happiness, pleasure,** anger, disgust, fear, pain, sadness, surprise	5 male 5 female—prof. actors and nonprof. actors	*f*_o_, SR, Power, Waveforms, Spectr
16	Belyk & Brown (2014)	SP	A	Motivational **(joy**, **gloating,** distress, resentment), Moral **(appreciation**, **gratitude,** reproach,), Aesthetic **(awe, pleasure,** disgust**,** terror)	10 male 22 female—nonprof. actors	*f*_o_, Int
17	Braun & Katerbow (2005)	SP	A	**Joy,** anger, fear, sadness	3 female 3 male—prof. actors	*f*_o_
18	Breitenstein et al. (2001)	SP	A	**Happiness,** anger, fear, sadness	1 female—prof. actor	*f*_o_, SR
19	Burkhardt & Sendlmeier (2000)	SP	ST	**Joy, happiness,** boredom, crying despair, fear, hot-cold anger, quiet sorrow	5 male 5 female	*f*_o_, Int, SR
20	Cahn (1990)	SP	ST	**Glad,** angry, disgusted, sad, scared, surprised	-	*f*_o_, Art., Pauses, Spectr, SR, glottal waveform
21	Carlson et al. (1992)	SP	ST	**Happy,** angry, sad	2 speakers	*f*_o_, SR
22	Chronaki et al. (2014)	NV	A	**Happiness,** anger, sadness	Taken from Maurege corpus, actors	Int
23	Corbeil et al., (2013)	SP, NV	I	**Happy/joyful**	1 female	*f*_o_, Amplitude, SR
24	Costanzo et al. (1969)	SP	A	**Love,** anger, contempt, grief, indifference	12 male 11 female—nonprof. actors	*f*_o_, Int, SR
25	Cowie & Douglas-Cowie (1996)	SP	A	**Happiness,** anger, fear, sadness	40 volunteers, nonprof. actors	*f*_o_, Int, SR, Energy, Spect
26	Dai et al. (2009)	SP	A	**Happy, interest,** hot anger, panic, sadness	3 male 5 female—prof. actors	Various features
27	Davitz (1964a)	SP	A	**Admiration, affection, amusement, cheerfulness, joy, satisfaction,** anger, boredom, despair, disgust, dislike, fear, impatience, surprise	4 male 4 female speakers	*f*_o_, Int, SR, Timbre
28	Davitz (1964b)	SP	A	**Affection, cheerfulness, joy, satisfaction,** anger, boredom, impatience, sadness	38 female, 23 male nonprof. actors	*f*_o_, Int, SR, Art, Rhythm, Timbre
29	Erickson et al. (2016)	SP	A	**Happy,** angry, sad	1 male 1 female—nonprof. actors	*f*_o_, Int, *F*_1_, SR
30	Fónagy (1978)	SP	A	**Coquetry, joy, tenderness,** anger, disdain, fear, longing, repressed anger, reproach, sadness	1 female—prof. actors	*f*_o_
31	Friend & Farrar (1994)	SP	A	**Happy,** angry	1 female	*f*_o_, Int, Spectr
32	Gårding & Abramson (1965)	SP	A	**Delighted surprise,** anger	5 speakers	*f*_o_
33	Gérard & Clement (1998)	SP	A	**Happiness,** irony, sadness	12 (6 children 3 adult)—nonprof. actors	SR, *f*_o_
34	Gobl & Chasaide (2000)	SP	ST	**Confident, content, friendly, happy, interested, relaxed, unafraid,** afraid, angry, bored, hostile, sad, stressed, timid	-	Int, Jitter, Formants, Spectr, Glottal Waveform
35	Goudbeek & Scherer (2010)	SP	A	**Amusement, elation, joy, interest, pride, pleasure, relief,** anxiety, cold anger, despair, hot anger, panic fear, sadness	5 male 5 female—prof. actors	*f*_o_, Int, SR, Shimmer, Spec, HNR
36	Hammerschimidt & Jürgens (2007)	SP	A	**Affection/tenderness, joyful surprise, voluptuous enjoyment/sensual satisfaction,** contempt/disgust, despair/lamentation, rage/hot anger	11 male 12 female—prof. actors	*f*_o_, Amplitude, SR, HNR, range
37	Higuchi et al. (1997)	SP	A, SN	**Gentle**, angry, hurried	1 male—prof. actor	*f*_o_ contour
38	Hirose et al. (2000)	SP	A	**Happiness,** anger, sadness	2 semi-prof. actors	*f*_o_, SR, Power,
39	House (1990)	SP	I	**Happy,** angry, sad	Taken from Gârding 1986	*f*_o_, Int
40	Huttar (1968)	SP	SN	**Bold, confident, happy, pleased, sure,** afraid, angry, sad, timid, unsure	1 male—nonprof. actors	*f*_o_, Int, SR
41	Iida et al. (2000)	SP	ST	**Joy,** anger, sadness	Male and female—nonprof. actors	*f*_o_, Int, SR
42	Iliou & Anagnostopoulos (2009)	SP	A	**Happiness,** anger, boredom, disgust, fear, sadness, surprise	5 male 5 female—prof. actors	*f*_o_, Formants, Energy
43	Iriondo et al. (2000)	SP	ST	**Desire, joy,** disgust, fear, fury, sadness, surprise	4 male 4 female actors	*f*_o_, Int, SR, Pauses, Spectr
44	Jiang et al. (2015)	SP	A	**Happiness,** anger, fear, sadness	2 male—lay actors	*f*_o_, Amplitude, SR
45	Jiang & Pell (2017)	SP, NV	A	**Confidence,** doubt	3 male 3 female—lay experience in acting or public speaking	*f*_o_, Int, SR, HNR, Jitter, Shimmer, Pause
46	Jo et al. (1999)	SP	ST	**Happy,** afraid, angry, sad	1 speaker	SR, *f*_o_
47	Johnstone & Scherer (1999)	SP	I	**Happy,** anxious, bored, depressed, irritated, tense	36 male—nonprof. actors	*f*_o_, Int, Spectr, Jitter, Glottal Waveform
48	Juslin et al. (2017)	SP	A, SN	**Happiness,** anger, sadness	Samples from 23 sources	88 features from GeMAPs
49	Juslin & Laukka (2001)	SP	A	**Happiness,** anger, disgust, fear, sadness	4 male 4 female—7 prof. actors, 1 semiprof. actor	*f*_o_, int, formant, energy, sr, pause
50	Jürgens et al. (2011)	SP	A, SN	**Joy,** anger, fear, sadness	21 male 21 female—31 prof. actors, 10 drama students, 1 prof. singer	*f*_o_, Amplitude
51	Jürgens et al. (2015)	SP	A, SN	**Joy,** anger, fear, sadness	21 male 21 female—30 prof. actors, 11 acting students & 19 male 12 female—nonprof. actors	*f*_o_, *F*_1_, Int
52	Kaiser (1962)	SP	A	**Cheerfulness, enthusiasm, kindness,** disgust, grimness, sadness,	2 male 2 female subjects	SR, *f*_o_, *f*_o_ contour, Int, Formants, Spectr
53	Kao & Lee (2006)	SP	A	**Happiness,** anger, fear, sadness	2 male 2 female—drama students	*f*_o_, Power, Energy, Formant, Pauses
54	Kienast & Sendlmeier (2000)	SP	A	**Happiness,** anger, boredom, fear, sadness	3 male 3 female actors	Spectr, formants, Art.
55	Laukka et al. (2005)	SP	A	**Happiness,** anger, disgust, fear, sadness	4 male 4 female—7 prof. actors, 1 semi-prof actor	*f*_o_, Jitter, Int, Formant, HF
56	Laukka et al. (2016)	SP	A	**Happiness, interest, lust, pride, relief,** anger, contempt, disgust, fear, sadness, shame,	10 male 10 female from each of 5 cultures—prof. actors	Parameters included in the GeMAPs
57	Laukkanen et al. (1996)	SP	A	**Enthusiasm, surprised (positive),** anger, sadness	1 male 2 female—prof. actors	*f*_o_, Int, glottal waveform, subglottal pressure
58	Laukkanen et al. (1997)	SP	A	**Enthusiasm, surprised (positive),** anger, sadness	2 male 1 female	Glottal waveform, formants
59	Leinonen et al. (1997)	SP	A	**Admiring, astonished, content**, angry, commanding, frightened, naming, pleading, sad, scornful	8 male 8 female—nonprof. actors	SR, *f*_o_, *f*_o_ contour, Int, Spectr
60	Levitt (1964)	SP	A	**Joy,** anger, contempt, disgust, fear, surprise	25 male 25 female—nonprof. actors	*f*_o_, Spectr
61	Lieberman & Michaels (1962)	SP	ST	**Happiness, pompous,** boredom, confidential, disbelief, fear	6 male	*f*_o_, Int
62	Lima et al. (2013)	NV	A	**Achievement/triumph, amusement, pleasure, relief,** anger, disgust, fear, sadness	2 male 2 female—nonprof. actors	*f*_o_, Int, SR, Spectr, HNR
63	Lima et al. (2014)	NV	A	**Achievement/triumph, amusement, relief, pleasure,** anger, disgust, fear, sadness	4 male 4 female speakers from 2 different sources	*f*_o_, Int, SR, Spectr, HNR
64	Liscombe et al. (2003)	SP	A	**Confident, encouraging, friendly, happy, interested,** angry, anxious, bored, frustrated, sad	2 male 2 female—prof. actors	*f*_o_, amplitude
65	Luengo & Navas (2005)	SP	A	**Joy,** anger, fear, disgust, surprise, sadness	1 female—prof.	*f*_o_, energy, jitter, shimmer
66	Moriyama & Ozawa (2001)	SP	A	**Joy,** anger, fear, sorrow	1 male actor	SR, *f*_o_, Int
67	Mozziconacci (1998)	SP	A	**Joy,** anger, boredom, fear, indignation, sadness	2 male 2 female	SR, *f*_o_, rhythm
68	Nagasaki & Komatsu (2004)	SP	A	**Agreement,** disagreement, hesitation	1 male	*f*_o_, SR, Int, Voice quality
69	Paeschke et al. (1999)	SP	A	**Happiness,** anger, boredom, fear, sadness	7 speakers	*f*_o_
70	Paeschke & Sendlmeier (2000)	SP	A	**Happiness,** anger, boredom, fear, sadness	5 male 4 female—prof. actors	*f*_o_
71	Pajupuu et al. (2015)	SP	A	**Joy,** anger, sadness	1 female	*f*_o_, Int, SR
72	Patel et al. (2011)	SP	A	**Joy, relief,** hot anger, panic fear, sadness	5 male 5 female—prof. actors	CQ, H1-H2, Leq, Shimmer, HNR, Jitter, Pulse Amp, *f*_o_ mean, Alpha, NAQ
73	Pell (2001)	SP	A	**Happy,** angry, sad	5 male 5 female—nonprof. actors	SR, *f*_o_
74	Pell et al. (2009)	SP	A	**Happiness, positive surprise,** anger, disgust, fear, sadness	2 male 2 female from each of 4 languages—nonprof. actors	SR, *f*_o_
75	Pell et al. (2015)	NV, SP	I	**Happiness,** anger, sadness	4 male 6 female & 5 female 5 male speakers	*f*_o_, Int
76	Pereira & Watson (1998)	SP	A	**Happiness,** cold anger, hot anger, sadness	1 male 1 female actors	*f*_o_, RMS
77	Petrushin (1999)	SP	A	**Happiness,** anger, fear, sadness	30 nonprof. actors	*f*_o_, *F*_1_, *F*_2_, Energy, SR
78	Pollerman & Archinard (2002)	SP	A	**Joy,** anger, sadness	30 male 6 female—nonprof. actors	*f*_o_, Voiced energy range
79	Rao et al. (2013)	SP	A	**Happiness**, anger, disgust, fear, sadness, sarcasm, surprise	5 male 5 female—prof. actors	SR, *f*_o_, Energy
80	Sauter et al. (2010)	NV	A	**Achievement, amusement, contentment, relief, pleasure, triumph,** anger, disgust, fear, sadness, surprise	2 male 2 female—nonprof. actors	*f*_o_, Int, Spectr
81	Scherer (1972)	SP	ST	**Elation, happiness, interest,** anger, boredom, disgust, fear, sadness, surprise	-	SR, *f*_o_, Int
82	Scherer (2013)	SP	A, I	**Happy,** sad	83 male nonprof. actors	energy, *f*_o_, spectr, and time domain
83	Scherer et al. (1991)	SP	A	**Joy,** anger, disgust, fear, sadness	2 male 2 female—prof. actors	*f*_o_, Int, SR, Spectr
84	Scherer et al. (2015)	SP, NV	A	**Joy, pride,** anxiety, anger, despair, fear, sadness	5 male 5 female—prof. actors	Tempo, SIL, Energy prop., Hammarberg Index, Spectral flatness, HNR, Jitter, Shimmer
85	Scherer & Oshinsky (1977)	SP	ST	**Happiness,** anger, boredom, disgust, fear, sadness, surprise	-	SR, *f*_o_, Int, Attack, Spectr
86	Seppänen et al. (2003)	SP	A	**Happiness/joy,** anger, sadness	8 male 6 female—prof. actors	*f*_o_ and various features
87	Skinner (1935)	SP	I	**Joy,** sadness	9 male 10 female—prof. actors	*f*_o_, Int, Spectr
88	Sobin & Alpert (1999)	SP	A	**Joy,** anger, fear, sadness	31 female	SR, pauses, *f*_o_, Int
89	Soderstrom et al. (2017)	NV	A	**Relief, triumph**	1 male & 2 male 2 female speakers	SR, Amplitude
90	Stibbard (2001)	SP	SN	**Happiness,** anger, disgust, fear, sadness	Samples taken from EISP data	Various features
91	Szameitat et al. (2009)	NV	A	**Joyful, tickling, schadenfreude,** Taunt	3 male 5 female -prof. actors	Many
92	Sztahó et al. (2011)	SP	SN	**Happy,** angry/nervous, sad	-	*f*_o_, Int, Mel-frequency, copstral coeff
93	Tanaka & Campbell (2011)	NV	SN	**Polite, mirthful**	5 male 5 female volunteers, nonprof. actors	*f*_o_, Formants, duration (spectral features)
94	Thompson & Balkwill (2006)	SP	A	**Joy,** anger, fear, sadness	Volunteers, nonprof. actors	*f*_o_, Int
95	Tischer (1995)	SP	A	**Affection, joy, love, tenderness, satisfaction, sexual pleasure,** anger, disgust, fear, rage, surprise, sadness, uncertainty, yearning	2 male 2 female—prof. actors	SR, Pauses, *f*_o_, Int
96	Toivanen et al. (2006)	SP	A	**Joy, tenderness,** anger, sadness	5 male 4 female, prof. actors	Jitter, shimmer, and SR
97	Trainor et al. (2000)	SP	A	**Comfort/love,** fear, surprise	23 female—nonprof. actors	SR, *f*_o_, *f*_o_ contour, rhythm
98	Trouvain & Barry (2000)	SP	SN	**Joy,** anger, fear, surprise	3 male speakers	*f*_o_, Int, Pause, Spect, tempo
99	van Bezooijen (1984)	SP	A	**Interest, joy,** anger, contempt, disgust, fear, sadness, shame, surprise	4 male 4 female—nonprof. actors	SR, *f*_o_, Int, Spectr, Jitter, Art
100	Viscovich et al. (2003)	SP	A	**Happy,** sad	10 male, 9 female—nonprof. actors	*f*_o_
101	Waaramaa et al. (2010)	SP	A	**Joy, tenderness,** anger, sadness	5 male 4 female—prof. actors	*f*_o_, equivalent sound level, alpha ratio
102	Wallbott & Scherer (1986)	SP	A	**Joy,** anger, sadness, surprise	3 male 3 female—prof. actors	SR, *f*_o_, Int
103	Wang et al. (2008)	SP	A	**Happiness,** anger, disgust, fear, sadness, surprise	Male female	*f*_o_, SR, energy
104	Whiteside (1999a)	SP	A	**Elation, happiness, interest,** cold anger, hot anger, sadness	1 male 1 female—nonprof. actor and prof. actor	*f*_o_, Int, SR, Formants
105	Whiteside (1999b)	SP	A	**Elation, happiness, interest,** cold anger, hot anger, sadness	1 male 1 female—nonprof. actor and prof. actor	*f*_o_, Int, Jitter, Shimmer
106	Yildirim et al. (2004)	SP	A	**Happiness,** anger, sadness	1 female—prof. actor	*f*_o_, SR, Formant, RMS energy, Spectr
107	Yuan et al. (2002)	SP	A	**Joy**, anger, fear, sadness	9 speakers	*f*_o_, pause
108	Zhang (2008)	SP	SN	**Joy**, anger, sadness	16 male 37 female speakers	SR, *f*_o_, *F*_1_, *F*_2_, *F*_3_, Energy, Jitter, Shimmer

*Note.* Positive emotion categories as used by the authors are marked in boldface. SP = speech prosody; NV = nonverbal vocalizations; A =acted; SN = spontaneous; I = Induced; ST = synthesized; *f*_o_ = fundamental frequency; Int = voice intensity; SR = speech rate; Dur = duration; nonprof = nonprofessional actors; prof = professional actors

Most of the studies focused exclusively on speech prosody (*n* = 92; 85%), a smaller number examined only nonverbal vocalizations (*n* = 11; 10%), and five studies (5%) included both. Among the studies providing information about speakers’ gender (*n* = 84; 78%), vocalizations were collected from only male (*n* = 12; 14%), only female (*n* = 9, 11%) speakers, or a combination of both (*n* = 63; 75%). Eighty-four studies used acted speech samples, in which speakers were asked to read carrier phrases in targeted emotional states for the construction of acted portrayals. These phrases included numbers or letters, nonsense utterances, meaningful utterances that were emotionally neutral in their verbal content, or masked verbal content. The number of speakers varied from 1 to 63. Most studies employed either professional or semi-professional actors (*n* = 35; 42%), or nonprofessional speakers (*n* = 20; 24%). Seven studies (8%) used both professionals and nonprofessionals, while some studies gave no information on the speakers’ acting experience (*n* = 21, 25%). Studies that did not use acted portrayals mostly tended to use spontaneous vocalizations (*n* = 14, 13%). In those studies, vocalization samples were selected from YouTube, TV series and shows, interviews, horse race commentaries, conversations, classroom discussions, radio interviews, and documentaries. Seven studies (6%) employed induction of positive emotions in an experimental setting, while 11 studies (10%) used synthesized or resynthesized vocalizations with modifications of acoustic parameters. Below, we discuss the positive emotion terms used in this research and provide an overview of the acoustic features.

## Terminology of positive emotions

**Fig. 1:**
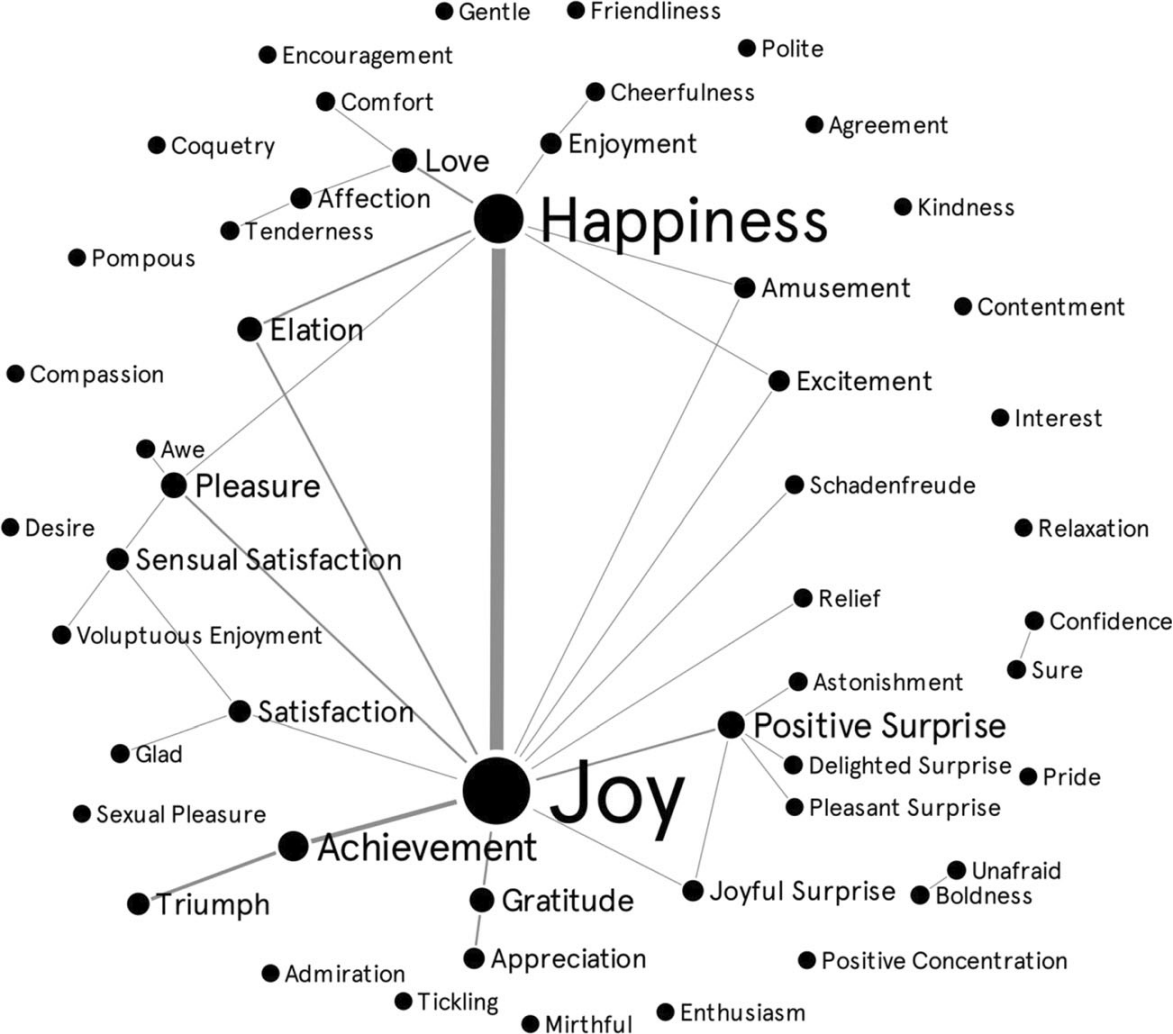
Different positive emotion terms used in research on acoustic features of positive emotions in the voice. Emotion categories are only linked if the material used for elicitation of two emotion categories was the same, or if the authors explicitly stated that the two categories were the same. For instance, if two studies used the same materials, but labelled them with different terms (e.g., happiness vs amusement), then a connection line was added between those terms. Similarly, if two emotion terms were explicitly treated as equivalent, such as with a slash mark (e.g., achievement/triumph), a parenthesis (e.g., elation [joy]), or used interchangeably in an article, then a connection line was created between the two emotion terms. Larger circles reflect terms used more often in connection with others. Thicker connections reflect more frequent connections

Table 1 presents all the emotion terms used in studies on the acoustic features of positive emotions. Among these, 52 different terms were used to refer to positive emotional states (see Fig. 1). Happiness was the most frequently used term (*n* = 53; 49%), followed by joy (*n* = 40; 37%). Other frequently used terms were interest (*n* = 10; 9%), pleasure (*n* = 10; 9%), amusement (*n* = 8; 7%), and relief (*n* = 7; 6%), while a substantial number of other terms were used in a small number of studies.

The disproportionately high use of the terms *happiness* and *joy* is likely to be due to two mutually compatible reasons. Firstly, many researchers have used the ‘basic emotion’ categories proposed by Ekman (see Ekman, 1992). Among the six most widely used categories of basic emotions (anger, disgust, fear, happiness/joy, sadness, and surprise), happiness/joy was long considered the only positive basic emotion. Even though other basic positive emotions have been suggested to be basic positive emotions (e.g., amusement: Keltner, 1995; interest: Izard, 2011; lust: Panksepp & Watt, 2011; pride: Tracy & Robins, 2008), the six basic emotions have been examined in many studies (see Table 1). Secondly, happiness and joy are conceptualized broadly. Some researchers have used happiness and joy to refer to a higher-order category encompassing other emotional states. For instance, joy has been defined as including gratitude, happiness, pleasure and exhilaration (Pajupuu, Pajupuu, Tamuri, & Altrov, 2015), or as a category including all positive emotions except amusement and sensual pleasure (Anikin & Persson, 2016).

The inconsistencies in what the terms joy and happiness are taken to mean across studies implies that the associated results likely involve inconsistencies. Indeed, in a review of more than 300 self-report measures tapping momentary distinct emotions, Weidman, Steckler, and Tracy (2017) drew attention to considerable ambiguity in the literature with respect to measurements of emotions. They highlighted overlap among emotion terms used in self-report scales, showing that positive emotions referring to the same emotional experience were measured with different words. For instance, researchers used many different words to measure joy, including *delighted*, *glad*, *joyful*, *lively*, *satisfied*, *happy*, *content,* and *enthusiastic*. Furthermore, different discrete positive emotions were sometimes measured with the same word. For instance, the word *happy* has been used to measure not only happiness and joy, but also excitement and schadenfreude.

In trying to explicate such inconsistencies, Fig. 1 maps the terminology used for emotion elicitation and/or specification in the studies in this review. It illustrates the frequency of connections of an emotion term with all of the other emotion terms overall (circle size), and the frequency of connections between two specific terms (line thickness). The graph is created with a Web-based platform, Graph Commons (graphcommons.com), which is a tool that visually disentangles complex relationships in data networks. A dynamic version of Fig. 1 is available at https://graphcommons.com/graphs/a85e068b-1f6f-44ab-8fa7-2621ba1f2971; this allows users to select data points or distinct positive emotion terms, showing their connections with other terms. As Fig. 1 shows, 35 different links were found between distinct positive emotion terms. Most frequently, happiness and joy were linked with each other or with other emotion terms: happiness was linked with seven, and joy with 12 other emotion terms. Considering the previously mentioned review of Weidman et al. (2017), one possibility is that researchers may have used different positive emotion terms, but actually measured happiness/joy (i.e., materials measuring happiness/joy were used but the elicited emotions were labelled with other positive emotion terms). They may also have used the terms happiness/joy, but in fact may have measured other positive emotions (i.e., materials measuring different positive emotions were used, but the elicited emotional states were labelled as happiness/joy). We return to this issue in the section Operationalizations, Design Features, and Recommendations for Future Research, where we make suggestions for how to address this issue in future research.

## Acoustic parameters of positive emotions

The measurement of acoustic parameters in emotional vocal expressions has focused on parameters in three domains: frequency (e.g., fundamental frequency, formant frequencies), amplitude (e.g., intensity), and duration (e.g., *speech rate*). To identify acoustic features in these domains that may relate to emotions, the source-filter theory (Fant, 1960; Titze, 1994) has been considered particularly helpful because it allows for relating the acoustics of vocalizations to changes in the producer’s physiological state (Briefer, 2012; Scherer, 1986). Below, we briefly introduce the source-filter theory of vocal production and then outline common acoustic features.

### Source-filter theory

**Fig. 2:**
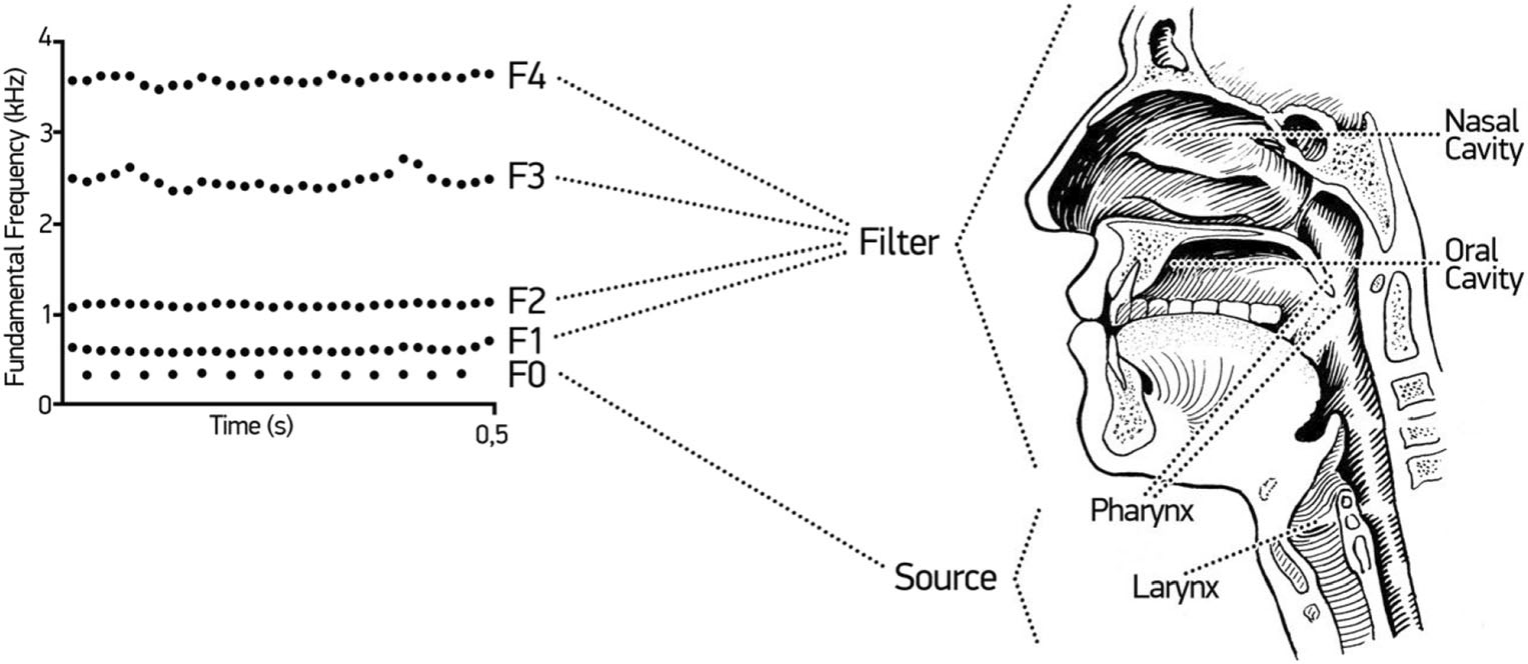
The source-filter framework of vocal production. Left: Spectrogram of a vocalization of the vowel /a/ illustrating *f*_o_ (*fundamental frequency*), and the first four *formant frequenciesF*_1_, *F*_2_, *F*_3_, *F*_4_. Right: Schema of the approximate locations of the vocal organs involved in the source and filter. Oscillation of the vocal folds in the larynx produces a source sound which determines the *fundamental frequency* (*f*_o_) of the vocalization. Then the sound is filtered through the vocal tract, which determines the *formant frequencies* (*F*_1_–*F*_4_)

The study of vocalizations in both humans and other mammals routinely applies the source-filter framework of vocal production, as illustrated in Fig. 2. The ‘source’ is located in the larynx and generates vocalizations. The air flow exhaled from the lungs oscillates the vocal folds, and the basic rate of vocal fold oscillation specifies the *fundamental frequency*. The sound waves produced by this oscillation travels though the pharynx—that is, the oral and nasal cavities that comprise the vocal tract. In this process, the vocal tract filters the sound, amplifying certain frequencies and attenuating others, thereby producing resonant frequencies called *formants*. These amplified and attenuated frequencies are determined by many factors, including the position of the tongue and the size and shape of the cavity. For example, a tongue positioned at the roof of the mouth produces different filtering effects—and consequently different sounding vocalizations—than a tongue positioned at the back of the teeth. An important feature of the source-filter framework is that the source and the filter can be controlled independently from each other; relevant to the present review, acoustic features relating to source and filter might compose different profiles for distinct emotional states.

## Common acoustic parameters

Table 2 shows definitions of common acoustic features and their perceptual correlates. The frequency of the first sinusoidal component is called *fundamental frequency*, or *f*_o_*.* It is the lowest frequency in a resonating system. It is determined by the rate of vocal fold (‘source’) vibration and is measured in Hertz, which refers to number of cycles completed per second. Its auditory correlate is the perceived pitch of the sound. *Formant frequencies* (e.g., *F*_1_*, F*_2_*, F*_3_) are the acoustic resonances of the vocal tract. As a speaker talks, for example, they change the shape of the vocal tract, which results in a variable acoustic ‘filter’. This allows more acoustic energy at certain frequencies, which are called *formant frequencies*. *Amplitude* refers to the air pressure in the wave, and is related to the amount of energy it carries. The perceptual correlate of amplitude is loudness. *Voice intensity* is energy through a unit area, such as square meter of air every second. Thus, as the *amplitude* of a sound wave increases, the *voice intensity* also increases. For illustration purposes, vocalizations with different *f*_o_ and *amplitude* levels are available at https://emotionwaves.github.io/acoustics/. *Speech rate* refers to a temporal aspect of vocalizations relating to the number of elements (e.g., syllables or words) per time unit (e.g., seconds or minutes). *Speech rate* can also be measured as the overall duration of an utterance if the utterance structure is determined a priori (e.g., how long it takes to say a given word).

**Table 2.** Common acoustic parameters and their definitions

Acoustic Parameter	Perceptual Correlate	Definition
*f*_o_ (fundamental frequency)	Pitch	Lowest periodic cycle of the acoustic signal
*F*_1_, *F*_2_ (formant frequencies)	Voice quality	Concentration of acoustic energy around first and second formants
Intensity and amplitude	Loudness	Measures of energy in the acoustic signal
Speech rate	Velocity of speech	Number of complete utterances or elements produced per time unit
Jitter	Pitch irregularity	Frequency instability of *f*_o_
Shimmer	Loudness irregularity	Amplitude instability of *f*_o_
Spectral Energy	Timbre	Relative energy in different frequency bands
Glottal waveform	Voice quality	The time of airflow between the vocal folds and the time glottis is closed for each vibrational cycle
HNR (harmonics to noise ratio)	Voice quality	Mean ratio of quasi-periodic to non-periodic signals across time segments

*Note.* Though parameter names may differ from those in the original studies, they correspond to the definitions given

In addition to *pitch*, *loudness*, and temporal aspects of vocal expression, *voice quality* is an important dimension of the voice source. *Voice quality* is the perceptual correlate of the pattern of energy distribution in the acoustic spectrum (e.g., representation of the amount of vibration at each frequency; Scherer, 1986). It is used to refer to features such as hoarseness, breathiness, harshness, and creakiness (also called vocal fry) of the voice, and is measured using *jitter, shimmer, glottal waveform*, and *harmonics-to-noise ratio* (*HNR*). *Jitter* and *shimmer* reflect variations from one cycle to the next: *Jitter* indicates the perturbation of *fundamental frequency*, while *shimmer* refers to *amplitude* perturbation. These measures are used as indices of voice stability. The normal voice has a small amount of instability that is caused by tissue and muscle properties. Large variations in perturbation result in voice instability that can be captured by *jitter* and *shimmer* measures. *Spectral energy* distribution is typically used to analyze the proportion of high-frequency energy. Specifically, it is indexed by the energy in the vocalization that is higher than a given cutoff value compared with the total acoustic energy. The voice sounds sharper and less soft as the proportion of high-frequency energy increases (Von Bismarck, 1974). The *glottal waveform* is the airflow between the vibrating vocal folds, the area known as the ‘glottis’. It is specific to individual phonation types and refers to the distinguishable characteristics of a voice. A feature related to *voice quality* is *HNR*. The *HNR* is a ratio quantifying the proportion of energy in the voice attributable to a periodic source. A lower value reflects a noisier vocalization, whereas a higher value reflects a more tonal sound.

## The current approach

**Fig. 3:**
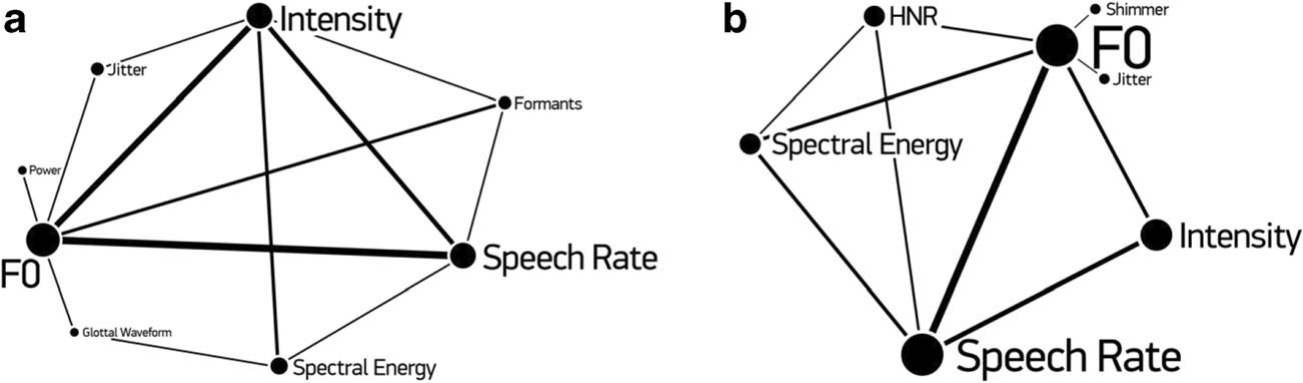
Acoustic features used at least in two separate publications. **a** Frequently used acoustic parameters involved in comparisons of individual positive emotions in comparison to neutral vocalizations. **b** Frequently used acoustic parameters involved in comparisons of acoustic features across several positive emotions. The size of each circle refers to the frequency of use of that type of acoustic feature; the thickness of a connection line between two acoustic features represents the frequency of inclusion of these features together in the same study. The larger the size of the circle, the more frequently a given feature has been studied; the thicker the connection line, the more frequently two acoustic features have been studied together

The current review aims to establish acoustic patterns of positive emotion(s) in speech prosody and nonverbal vocalizations. We employ a descriptive analysis with a comparative approach to identify the acoustic patterns of discrete positive emotions. This is necessary because information regarding the exact settings of the extraction tools and computation of acoustic parameters is often lacking, making it impossible to conduct statistical comparisons of quantitative data across studies. Furthermore, research attempting to determine acoustic features of positive emotions have used different emotion elicitation methods, different numbers of speakers with different level of acting experience, and have varied in terms of speaker gender (see Table 1). Moreover, studies to date have varied considerably in the types of acoustic parameters they have included. Figure 3 presents the most frequently used acoustic features.

Following the approach described above, common acoustic features used in studies comparing at least one positive emotion to a neutral voice (see Fig. 3a; click https://graphcommons.com/graphs/cc0605c9-c9c8-4c10-a1bb-34725f9d5f9d for an interactive map), or across positive emotions (see Fig. 3b; click https://graphcommons.com/graphs/5bb0001b-1049-488d-9396-3eaf2384c7fe for an interactive map) are illustrated. To review potential systematicities in acoustic features, we conducted two types of comparisons, both within study. In the first, we included studies comparing acoustic patterns of at least one positive emotion to a neutral state. Some studies did not include a neutral category, but instead computed an overall mean across all emotions as a baseline. Previous reviews have tended to use such variable reference points (e.g., Murray & Arnott, 1993). We exclusively examined studies that included a neutral baseline, since a baseline computed from the other conditions is determined by the specific set of emotions included in a given study. Our approach differs in a further aspect from those employed in previous reviews on acoustics of emotions (e.g., Juslin & Laukka, 2003). Previous reviews have used broad categories such as high, medium, and low to describe levels of acoustic features, mainly based on the authors’ interpretations. We sought to avoid any interpretation of what constitutes high, medium, or low levels of acoustic features, and instead we only included studies providing acoustic data allowing us to directly compare features. By summarizing findings from such studies, we conclude with the most likely vocal indicators of positive emotions.

In the second comparison, we review studies that included more than one positive emotion category. These studies thus enabled a direct comparison of acoustic features *across* positive emotions.

## Results

### Acoustic features of positive emotions compared with neutral baseline

Twenty-six of the 108 studies (24%) investigated acoustic features of at least one positive emotion in comparison with a neutral condition. These are presented in Table 3.

**Table 3.** Changes in acoustic parameters of positive emotions compared with neutral vocalizations

Category	Effect direction	Happiness	Joy	Elation	Interest	Tenderness	Pleasure	Positive surprise	Pride	Relief	Lust	Satisfaction
F0 mean	>	2a,2b,7,14,44a,44b,49a,49b,53,56,69,74a,74b,74c,74d,79a,79b,100a,100b,105b*,104b*	7,17a,17b,17c,17d,17e,17f,71,99,101a,101b	104b,105b	56**,**99,104b,105b			74a,74b,74c,74d	56	56		
	<	104a*,105a*		104a,105a	104a,105a	101a,101b					56	
F0 variability	>	2a,2b,14,**15**,18a,18b,47 49a,49b,53,79a,44a,44b,100a,100b,104a,105a,105b	99,101b	104a,104b,105a,105b	99,104a,104b,105a,105b	101b	**15**					
	<	79b,104b	101a			101a						
F0 range	>	7,**15**,39,44a,44,69,74a,74b,74c,74d	5,7,17a, 17b,17d, 17e,17f,19,71,99,101b		99	101b	**15**	74a,74b, 74c,74d				5,7
	<	14,47	17c,101a			101a						
F1 mean	>	49a,49b, 104a,104b	101b	104a,104b	104a,104b	101a						
	<		101a			101b						
F2 mean	>	104a,104b	101a,101b	104a	104a	101a101b						
	<			104b	104b							
Voice intensity mean	>	2a,2b,8,38,39,49a,49b,53,56,79a,79b,104a, 104b,105a,105b		104a,104b,105a,105b	56,104a,105a,105b				56	56		
	<	44a,44b	71								56	
Voice intensity variability	>	2,14,**15**,44a,49a,53,104a,105a,1105b		104b,105a,105b	105a,105b		**15**					
	<	44b,104b		104a	104a,104b							
Voice intensity range	>	14,44b										
	<		71									
Speech rate	>	14,38,49b,74b,104b	71,19	104b	99,104b		**15**					
	<	2a,2b,**15**,18a,18b,21,44b,49a,74a,74c,74d,79a,79b,104a,	99,101a,101b	104a		101a, 101b						
Jitter	>	47,49a,49b,105a		105a,105b	105a,105b							
	<	105b										
Shimmer	>	105a		105a,105b	105a,105b							
	<	105b										
HNR	>	56			56				56	56		
	<										56	

*Note.* The numbers correspond to the codes given in Table 1. ‘>’ indicates that parameter value is higher/faster in the positive emotion than neutral condition; ‘<’ indicates that parameter value is lower/slower in the positive emotion than neutral condition. Studies using nonverbal vocalizations are marked in boldface, others used speech prosody. * = used same male or female actor in the same project. 2a = Arabic; 2b = English; 17a = German female; 17b = Japanese female; 17c = English female; 17d = German male; 17e = Japanese male; 17f = English male; 18a = German; 18b = English; 44a = Hindi; 44b = English; 49a = high intensity; 49b = low intensity; 74a = English; 74b = German; 74c = Hindi; 74d = Arabic; 79a = female; 79b = male; 100a = female; 100b = male; 101a = female; 101b = male; 104a = female; 104b = male; 105a = female; 105b = male

#### Happiness

Most of this research studied happiness, with a shift towards higher *f*_o_*mean*, *variability*, and *range*, and higher *voice intensity mean* and *variability* for happy compared with neutral vocalizations. Each of these patterns of results was supported by between five and 14 studies, and no more than two studies found an opposite pattern of results. Thus, these parameters can be considered the clearest acoustic indicators of vocal expressions of happiness. Furthermore, *F*_1_ and *F*_2_*means* were consistently found to be higher in happy as compared with neutral vocalizations, although these features were measured in fewer studies. These first two *formants*, *F*_1_ and *F*_2_, are important acoustic parameters in human speech, and alterations result from the length and shape of the vocal tract being modified by the vocal articulators (Fant, 1960). For instance, the size of the oral and pharyngeal cavity can be modified by the articulators such as tongue, lips, and soft palate. Thus, constriction of the vocal tract in different places creates different patterns of change in *F*_1_ (around 500 Hz) and *F*_2_ (around 1500 Hz).

By contrast, results on *speech rate* are inconsistent: happy vocalizations were characterized by slower *speech rate* in nine studies, whereas five studies found happy vocalizations to have increased *speech rate*. Furthermore, some of the *speech rate* findings varied based on the gender of the speaker, emotional intensity of expressions, and the language of the recorded speech. Finally, limited evidence suggests that energy-related features like *voice intensity range*, and *HNR*, as well as *jitter*, are all higher in happy compared to neutral vocalizations. However, the evidence for these features is tentative, as it is based on only a few studies. It is notable that the findings on *f*_o_*variability* and *range*, *voice intensity variability*, and *speech rate* were similar in a study of nonverbal vocalizations (Belin, Fillion-Bilodeau, & Gosselin, 2008) to those on speech prosody (e.g., Al-Watban, 1998; Jiang, Paulmann, Robin, & Pell, 2015).

#### Joy

In the case of joy, all of the six studies that examined *f*_o_ mean found joyful vocalizations to be associated with an increase in *f*_o_*mean*. Seven studies found an increase in *f*_o_*range* for joyful vocalizations, whereas results for two studies varied based on the gender of the speaker and the language of the recording. All of the studies on joy in the voice examined speech prosody.

#### Other positive emotions

In addition to happiness and joy, researchers have investigated acoustic parameters of several other distinct positive emotions as compared with neutral vocalizations. For interest, *f*_o_*mean* has been found to be higher in four studies (but primarily for male speakers). Increases in *f*_o_*variability* (three studies) and *voice intensity mean* (three studies) have also been found. Notably, the pattern of results did not differ between nonverbal vocalizations and speech prosody. In the case of elation, *f*_o_*mean* has been found to be higher compared to neutral vocalizations, but only for male vocalizations (two studies). Furthermore, *f*_o_*variability* was higher (two studies), as was *voice intensity mean* (two studies) for elated as compared with neutral vocalizations. For satisfaction, a higher *f*_o_*range* has been supported in two studies. Unfortunately, evidence for other acoustic feature changes, as well as evidence relating to other positive emotions compared with neutral vocalizations, comes from single studies. Among these, tenderness and lust stand out in that they seem to be associated with a decrease in *f*_o_*mean*. While results for elation, tenderness, pride, relief, and lust were from studies using only speech prosody, results for pleasure were from studies using only nonverbal vocalizations.

Because of the lack of research into many positive emotions, knowledge on the acoustic patterns of most positive emotions presented in Table 3 is sparse. Therefore, we next examined studies that compared several positive emotion categories.

### Comparisons of acoustic features across positive emotions

Findings relating to the 20 studies (19%) that investigated acoustic features of multiple positive emotions are presented in Table 4. When compared with other positive emotions, *f*_o_*mean* was higher for joy, amusement, interest and relief, moderate for pleasure and contentment, and lower for lust and admiration (11 studies). *Voice intensity* mean was higher for joy, amusement, interest, and relief, moderate for contentment and pleasure in speech prosody (nine studies). *Speech rate* also yielded clear differences across the positive emotions. *Speech rate* was faster for pride, relief, and joy than it was for interest, and it was slower for pleasure, contentment, and admiration (10 studies).

**Table 4.** Changes in acoustic parameters of positive emotions compared with each other

Emotions compared		*f*_o_ (*M*)	*f*_o_ variability	*f*_o_ Range	*F*_1_ (*M*)	*F*_2_ (*M*)	Voice int. (*M*)	Voice int. variability	Speech rate	Jitter	Shimmer	HNR
Jo	af	>:28					>:28		>:28			
	am	>:35					>:35		>:35		<:35	
	ch	>:28					>:28		>:28			
	in	>:35	>:35, 99	>:99			>:35	>:35	>:35, 99			
		<:99									<:35	
	pl	>:35	>:35				>:35	>:35	>:35			
											<:35	
	pr	>:35	>:35				>:35	<:35	<:35	>:35		
											<:35	
	re	>:35*, 72*	>:35				>:35					>: 72
								<:35	<:35	<:72	<:35,72	
	sa	>:28					>:28		>:28			
		<:7		<:7								
	sc											
		<:**91b**			<:**91a**,**91b**							
	ti											
		<:**91a**,**91b**				<:91a						
	te	>:101a,101b	>:101a	>:101a	>:101a				>:101a,101b			
			<:101b	<:101b	<:101b	<:101a,101b						
Ha	el	>:104a,105a, 105b	>:81,104a, 105b			>:104b	>:104a,105a	>:81,104a, 105a	>:104b			
		<:9,104b	<:9,104b,105a		<:104a,104b	<:104a	<:9,104b,105b	<:104b,105b	<:9,104a	<:105a,105b	<:105a, 105b	
	in	>:56,104a,105a, 105b	>:9,81,104a, 105a		>:104a	>:104b	>:56,104a, 105a,105b	>:81,104a, 105a,105b	>:9,104b			
		<:9,104b	<:104b,105b	<:104	<:104b	<:104a	<:8,104b	<:104b	<104a	<:105a,105b	<:105a, 105b	<:56
	lu	>:56					>:56					>:56
	pl		**>:15**	**>:15**				**>:15**				
	pr	>:56	>:9				>:56		<:9			>:56
		<:9					<:9					
	re	>:56					>:56					>:56
	su	<:74a,74b,74c, 74d		<:74a,74b, 74c,74d					>:74b			
									<:74a,74c,74d			
In	am	<:35	<:35				<:35	<:35	<:35		<:35	
	el	>:104b,105a	>:81,104b, 105b		<:104a,104b	<:104a,104b	>:104b	<:81,104a, 104b,105a, 105b	>:104a,104b		>:105b	
		<:9,104a,105b	<:9,104a, 105a				<:9,104a, 105a,105b		<:9,105a,105b	<:105a,105b	<:105a	
	lu	>:56					>:56					>:56
	pl	>:35					>:35	>:35	>:35		<:35	
	pr	>:9,56	>:9,35				>:9,56			>:35		
							<:35	<:35	<:9,35		<:35	>:56
	re	>:35,56					>:56					>:56
			<:35				<:35	<:35	<:35		<:35	
Pl	ac		>:**80**	>:**80**								>:**63**
		<:**62**,63*,**80**	<:**62**,**63**	<:**62**,**63**			<:**62**,**63**,**80**	<:**62**, **63**	<:**62**,**63**,**80**			<:**62**
	am		>:**80**				>:**62**,**63**,**80**	>:**80**	>:3b,**80**		>:35	>:**62**,**63**
		<:35,**62**,**63**,**80**	<:3a,3b,35, **62**,**63**	<:**62**,**63**,**80**			<:35	<:35,**62**,**63**	<:3a,35,**62**,**63**			
	co	>:**80**	<:**80**	<:**80**			>:**80**		>:**80**			
	pr		>:35							>:35	>:35	>:**62**,**63**
		<:35					<:35	<:35	<:35			
	re		>:**80**	>:**80**			>:**62**,**63**,**80**	>:**80**	<:35,**62**,**63**,**80**		>:35	>:**62**,**63**
		<:35,**62**,**63**,**80**	<:35,**62**,**63**	<:**62**,**63**			<:35	<:35,**62**,**63**				
Re	ac	>:**62**,**63**	>:**62**					>:**62**,**63**	>:**62**,**63**,**80**			
		<:**80**	<:**63**,**80**	<:**62**,**63**,**80**			<:**62**,**63**,**80**	<:**80**				<:**62**,**63**
	am	>:**62**,**63**	>:**62**				>:**62**,**80**	>:35,**62**,**80** <:**63**	>:35,**62**,**63**,**80**			
		<:35,**80**	<:35,**63**,**80**	<:**62**,**63**,**80**			<:35,**63**					<:**62**,**63**
	co	>:**80**	<:**80**	<:**80**			>:**80**	<:**80**	>:**80**			
	lu	>:56					>:56					>:56
	pr		>:35							>:35	>:35	>:56
		<:35,56					<:35,56		<:35			
Am	ac		>:**62**,**63**,**80**	>:**62**,**80**				>:**63**	>:**62**,**63**			
		<:**62**,**63**,**80**		<:**63**			<:**62**,**63**,**80**	<:**62**,**80**	<:**80**			<:**62**,**63**
	co	>:**80**		>:**80**								
			<:**80**				<:**80**	<:**80**	<:**80**			
	el											
		<:9	<:9				<:9		<:9			
	pr	>:35	>:35				>:35			>:35	>:35	
								<:35	<:35			
Pr	af	>:28,36b	>:36a,36b				>:28,36b	>:36b	>:28,36a,36b			>:36b
		<:36a						<:36a				<:36a
	lu	>:56					>:56					>:56
Sa	ch											
		<:28					<:28		<:28			
	jo su								>:36a; <:36b			>:36a, 36b
		<:36a,36b	<:36a,36b				<:36a,36b	<:36a, 36b				
Af	ch	<:28					<:28		<:28			
	jo su								>:36a,36b			>:36a, 36b
		<:36a,36b	<:36a,36b				<:36a,36b	<:36a,36b				
Co	ac		>:**80**	>:**80**					>:**80**			
		<:**80**					<:**80**					
	ad	>:59		>:59					>:59			

*Note.* The numbers correspond to the codes given in Table 1. ‘>’ indicates that parameter value is higher/faster in the positive emotion listed on the left than the right; ‘<’ indicates that parameter value is lower/slower in the positive emotion listed on the left than the right. Studies using nonverbal vocalizations are marked in boldface, others used speech prosody. * = used partially same stimuli with another study; ac = achievement; ad = admiration; af = affection; am = amusement; ch = cheerfulness; co. = content; el = elation; ha = happiness; in = interest; jo su = joyful surprise; lu = lust; pl = pleasure; re = relief; ti = tickling; sc = schadenfreude; sa = satisfaction; su = positive surprise; HNR = Harmonics to Noise Ratio; 3a = spontaneous; 3b = acted; 36a = female; 36b = male; 74a = English; 74b = German; 74c = Hindi; 74d = Arabic; 91a = female; 91b = male; 101a = female; 101b = male; 104a = female; 104b = male; 105a = female; 105b = male

For several measures, results were markedly different for nonverbal vocalizations and speech prosody. The *voice intensity mean* of pleasure and contentment was higher than that of amusement in nonverbal vocalizations, but lower for speech prosody. Relief vocalizations had lower *voice intensity mean* than did interest, but for speech prosody, relief had higher *voice intensity* than did interest. Lastly, although more empirical research is required, it is possible to interpret *shimmer* and *HNR* findings. *Shimmer* was higher for pleasure, moderate for interest, and lower for joy (two studies). *HNR* was higher for pleasure and interest, moderate for relief and pride, and lower for lust (three studies).

### Effect of type of vocalizations on acoustic patterning

Speech prosody differs from nonverbal vocalizations in how they are produced. It has been suggested that nonverbal vocalizations are more strongly affected by physiological changes and their effects on the vocal organs than are prosodic expressions (Laukka et al., 2013), which might result in different patterns of acoustic features (e.g., Bachorowski, Smoski, & Owren, 2001). Furthermore, compared with speech prosody, nonverbal expressions do not require precise movements of articulators, because they are not constrained by linguistic codes (Scott, Sauter, & McGettigan, 2009).

Our results point to some differences in the acoustic features characterizing some emotions when expressed by speech prosody as compared with nonverbal vocalizations. For example, for nonverbal vocalizations, pleasure was *louder* than amusement and relief, whereas for speech prosody, pleasure was *quieter* than amusement and relief. These findings point to the importance of differentiating between nonverbal vocalizations and speech prosody because the patterns of results are sometimes different to the point of being opposite.

### Acoustic patterns associated with arousal

In previous studies, *pitch* and *loudness* have been considered key indicators of physiological arousal (e.g., Banse & Scherer, 1996; Scherer, 1986). For instance, *pitch* has been found to be higher in emotions like hot anger that are characterized by high levels of arousal, as compared with low arousal emotions like sadness (Patel, Scherer, Björkner, & Sundberg, 2011). In addition to *pitch* and *loudness* differences, under high arousal, the tempo of the sequence of phonatory and articulatory changes tends to be faster compared with low arousal states (Scherer, Sundberg, Tamarit, & Salomão, 2015).

Our findings are consistent with previous work on acoustic features associated with emotional arousal. For example, happiness, typically considered a state of high arousal (Scherer, 2003), had higher *pitch* and *loudness* as compared with neutral vocalizations. Similarly, joy and amusement, also considered high arousal positive emotions (e.g., Fredrickson, 1998), were higher in *pitch* and *loudness* than were pleasure and contentment, which are typically considered lower arousal positive emotions (e.g., Bänziger, Mortillaro, & Scherer, 2012). Furthermore, joy and pride, high arousal emotions (e.g., Cavanaugh, MacInnis, & Weiss, 2016), were characterized by higher *speech rate* when compared with pleasure and contentment, two low arousal emotions.

Our findings thus support the notion that *pitch* and *loudness* may reflect arousal, based on the evidence from studies including happiness, joy, and amusement. Furthermore, *speech rate* of high arousal positive emotions may be faster than *speech rate* of low arousal positive emotions. However, the arousal account does not capture variability in other acoustic features as well as systematic differences among a wide range of positive emotions other than happiness/joy/amusement.

### Listeners’ perception of vocal expressions of positive emotions

Most of the research included in Tables 3 and 4 used emotional stimuli enacted by actors (81%). Even though the use of actors is a popular method for researching acoustic parameters of positive emotions, it is not clear to what extent acted emotions are representative of expressions of genuine positive emotions (see Acted versus spontaneous expressions for a detailed discussion). Concerns about ecological validity is one of the reasons that studies using acted portrayals have included recognition studies. After listening to a vocal stimulus, listeners are typically asked to select which emotion they thought was expressed from a list emotion words. Generally, the percentage of correctly recognized stimuli is calculated per emotion and compared with the chance level, based on random guessing. Table 5 shows the studies (*n* = 20) that have reported recognition accuracy of positive emotion vocalizations. All of the studies found better than chance level recognition accuracy in recognition of vocally expressed positive emotions. Highest recognition rates were reported for amusement, achievement, relief, and pleasure, and lowest recognition rates were reported for elation and pride. Overall, the mean recognition rate in studies of nonverbal vocalizations (71.7%) was higher than that of speech prosody (60%). However, it is worth noting that data for most of the emotions are from studies of either only speech prosody or only nonverbal vocalizations.

**Table 5.** Listeners’ recognition rates (%)

Study No	Happiness	Joy	Elation	Interest	Amusement	Pleasure	Positive surprise	Pride	Relief	Lust	Achievement	Others
2	73(Arabic), 71(English)											
9	52		38	75				43				
**15**	60					59						
17		40*(male) 55*(female)										
19	61.9**	81**										
36							43<(accuracy)<87					43<(af), (en), (sa)<87
39	87											
44	66(English), 70(Hindi)											
49	51											
53	92.3**											
56	56			43				18	35	48		
59												34(ad), 72(as), 26(co)
**62**					95.9	85.9			86.3		77.7	
**63**					91	86.5			89		80	
69	(accuracy)>80											
71		75.4										
74	79.6(English) 59.6(German) 67.1(Hindi) 59.9(Arabic)						71.5(English) 68.8(German) 57.9(Hindi) 50.4(Arabic)					
**80**					79.5	65			86		77	46(co)
**91**		44										37(sc), 45(ti)
99		72		63								
Mean NV	60	44			88.8	74.1			87.1		78.2	46(co), 37(sc), 45(ti)
Mean SP	>67.6	64.7	38	60.3			62.1	30.5	35	48		34(ad), 72(as), 26(co)
Mean Total	>67.1	61.2	38	60.3	88.8	74.1	62.1	30.5	74.1	48	78.2	34(ad), 72(as), 36(co), 37(sc), 45(ti)

*Note.* The numbers correspond to the codes given in Table 1. Studies using nonverbal vocalizations are marked in bold, others used speech prosody. * = Based on stimuli produced by one speaker, full confusion matrix is not reported. ** = After best acoustic feature selection. ad = admiration; af = affection; as = astonishment; co. = content; en = enjoyment; sa = satisfaction; sc = schadenfreude; ti = tickling

## General discussion

### Summary of evidence

This article provides a comprehensive review of the acoustic features that characterize vocal expressions of positive emotions. Overall, past research has examined the acoustic features of positive emotions primarily by including a single category of happiness/joy and comparing it to negative emotions (see Table 1). Nevertheless, we were able to identify 26 studies reporting acoustic features of happiness/joy in comparison with a neutral state. We also identified 20 studies that reported acoustic features of a wide range of different positive emotions in comparison with each other. First, we reviewed research comparing any positive emotion with a neutral baseline. We found that *pitch*, *loudness*, and *formant* features are the clearest indicators of happiness in the human voice. In particular, when compared with neutral vocalizations, the voices of people who expressed happiness were higher across a range of measures: *pitch mean*, *variability*, and *range*, and *loudness mean* and *variability*, as well as the first two *formant means*. Because of limited empirical evidence, we were not able to draw clear conclusions for other acoustic features. However, based on the available findings, likely candidates are higher loudness range, *HNR*, and *jitter*. In the case of joy, higher *pitch mean* was the clearest indicator when compared with neutral vocalizations. Besides happiness and joy, only a few other positive emotions have been compared with neutral vocalizations. Among these, *pitch mean*, *pitch variability*, and *loudness mean* were higher when expressing interest or elation compared with neutral vocalizations. The acoustic features for other positive emotions were supported by only one study or were inconsistent (i.e., results indicating both increase and decrease for a given feature), and so further data are needed to yield reliable conclusions.

**Fig. 4:**
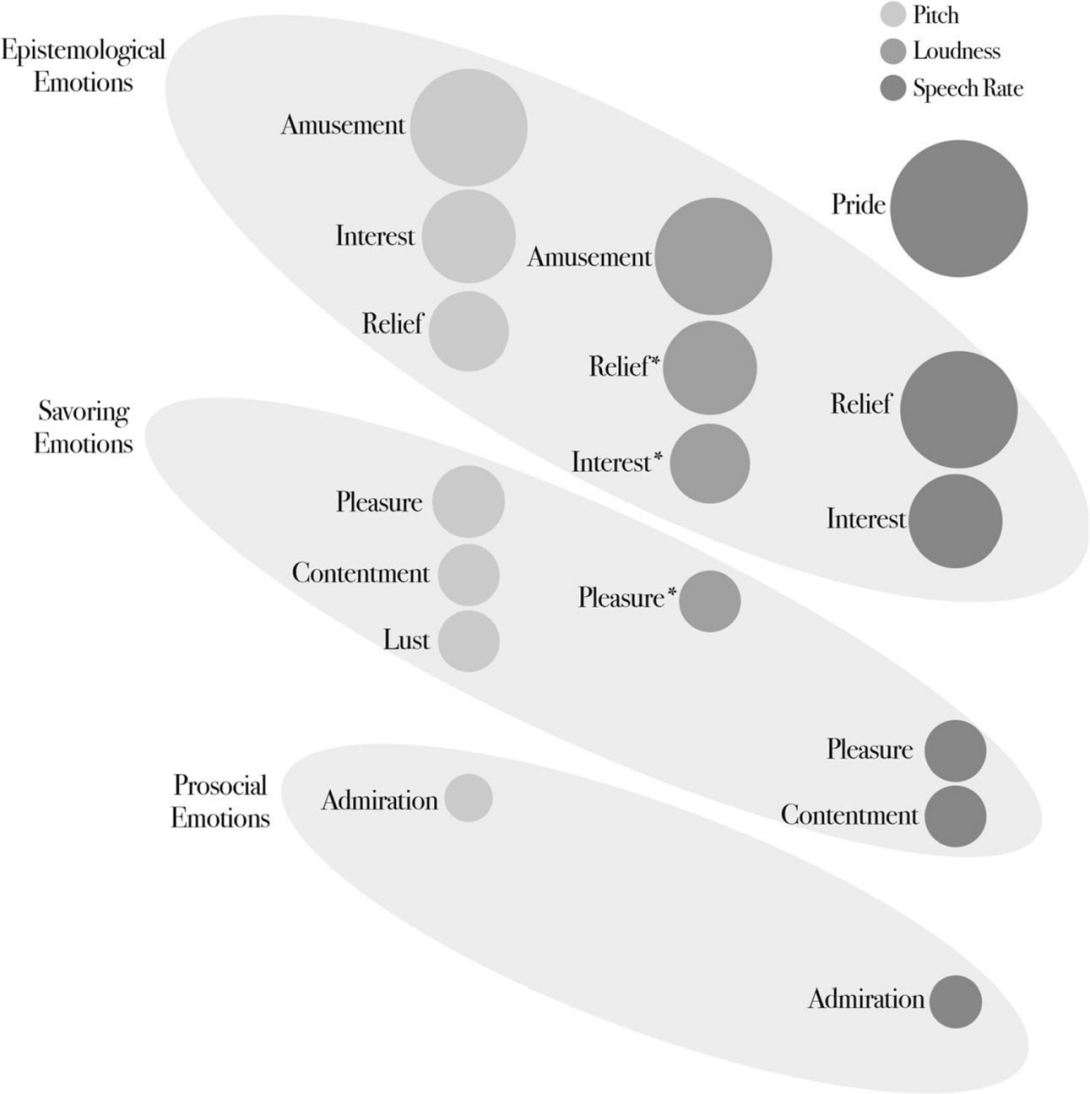
* = Only for speech prosody. Emotion families of positive emotions based on *pitch*, *loudness*, and *speech rate*. The larger the circle, the higher the related acoustic feature

Second, we reviewed research comparing acoustic features across different positive emotions. These findings highlighted differences in *pitch mean*, *loudness mean*, *speech rate*, and, to a lesser extent, *HNR* and *shimmer*. *Pitch* was found to be higher for epistemological emotions (amusement, interest, relief), moderate for savouring emotions (contentment, pleasure, lust), and lower for prosocial emotions (admiration; see Fig. 4). A similar pattern was found for *loudness*, which was higher for epistemological emotions (amusement, interest, relief) and lower for pleasure, a savouring emotion. *Speech rate* was faster for pride, and epistemological emotions (relief and interest), and slower for savouring emotions (pleasure and contentment) and admiration, a prosocial emotion. We also consider an alternative framework of emotional states, specifically evaluating whether an arousal dimension could explain variability in acoustic features between positive emotions. However, the arousal approach fails to account for variability in acoustic features other than *pitch* and *loudness*, and also fails to capture systematic differences among a wide array of positive emotions other than happiness/joy/amusement.

Our review differs in two major ways to previously published reviews of positive emotions in the voice (e.g., Juslin & Laukka, 2003; Murray & Arnott, 1993; Scherer, 2003). Firstly, we focused on acoustic patterns associated with positive emotions. For this purpose, we selected studies that provided a comparison with acoustic features of a neutral voice, in addition to those including several positive emotions. Previous reviews included studies using an overall mean across all emotions as a frame of reference, or broad categories (e.g., high, medium, low) to describe the level of acoustic features based on the authors interpretations. Here, we selected studies allowing us to compare actual acoustic data of an emotional voice with a neutral expression. Even though this is a strict criterion compared with other approaches, it is essential for conducting reliable within-study comparisons. Secondly, we included studies not only of speech prosody but also research on nonverbal vocalizations like laughs, sighs, and cheers. Previous reviews only focused on speech prosody and thus neglected nonverbal vocalizations which constitute an important nonlinguistic way of expressing emotions in the voice. In our review, we included a systematic analysis of differences and similarities of acoustic features associated with positive emotions across the two types of vocalizations. Notably, findings on acoustic features of happiness did not differ between nonverbal vocalizations and speech prosody. This provides a novel demonstration of consistency of acoustic features across different vocalization types used to express happiness. Furthermore, our results point to some differences in the acoustic features characterizing pleasure, amusement, and relief when expressed via speech prosody as compared with nonverbal vocalizations. Voices with pleasure were *louder* than were those with amusement and relief for speech prosody, but *quieter* for nonverbal vocalizations. These findings point to the importance of differentiating between nonverbal vocalizations and speech prosody because the patterns of results are sometimes different to the point of being opposite.

### Focus on source parameters

The source-filter framework (see Fig. 2) treats vocalizations as a combination of source energy and vocal-tract filtering; emotion-related effects can occur in both the source and the filter parts of the vocal production system (see, e.g., Scherer, 1986). In terms of differentiating between positive emotions, our review revealed differences mainly in source-related parameters. This reflects the fact that past research has focused primarily on *pitch* (*n* = 20, 100%), *loudness* (*n* = 16, 80%) and *speech rate* (*n* = 15; 75%). Filter related acoustic features such as *formant frequencies* and *energy distribution* have been more rarely considered in studies of positive emotions. Research suggests that filter related features, particularly *energy distribution* in the spectrum, might be important for differentiating emotional valence even between emotions of similar arousal level (e.g., Banse & Scherer 1996; Pollermann & Archinard, 2002; Waarama, Laukkanen, Airas, & Alku, 2010), whereas source-related parameters do not allow differentiation of valence, but do differentiate between discrete emotions (Patel, Scherer, Björkner, & Sundberg, 2011). However, more research measuring a large set of parameters including filter-related features is needed to obtain acoustic features for a larger set of discrete emotions. For instance, our results suggest that *shimmer* and *HNR* may be promising candidates for understanding acoustic features of different positive emotions. In addition, extending basic source-related measures will also be imperative for a better understanding of the acoustic patterns of (positive) emotions. Recently, an open-source measurement tool, GeMAPs (Eyben et al., 2016), for emotional voice analysis has been introduced to allow for a more standardized approach in the study of acoustics in relation to emotions in the human voice. The adoption of this tool could greatly expedite the accumulation of knowledge in this field.

### Operationalizations, design features, and recommendations for future research

It is worth noting that inconsistencies relating to some measures (see Tables 3 and 4) may reflect a lack of consistency in methodologies across studies. These methodological differences illustrate a wide range of approaches to studying emotions in the voice, which is a great asset. However, this variability also highlights the need to gain a deeper understanding of the role of operationalizations and design features in the vocal production of (positive) emotions. Next, we discuss operationalization of emotion, methods used for elicitation of emotions, and speaker samples used in research on emotional vocalizations.

#### Operationalizations of emotion, mood, and attitude

The studies included in this review have used the terms *emotion, mood,* and *attitude* inconsistently. Some researchers did not differentiate these concepts and used them interchangeably (e.g., Abelin & Allwood, 2000; Erickson, Zhu, Kawara, & Suemitsu, 2016; House, 1990), whereas others specifically used the term *mood* to refer to a target state (e.g., Bachorowski & Owren, 1995; Barrett & Paus, 2002; Lieberman & Michaels, 1962). These terms do not, in principle, refer to equivalent phenomena, however. Three main features have been proposed to distinguish emotions from moods and attitudes (e.g., Ekman & Davidson, 1994): (1) Emotions are evoked in reaction to a particular stimulus of major significance to the individual having the emotion. Emotions are therefore more sudden than are moods and attitudes. (2) Emotions have the potential to be more intense compared with moods and attitudes, which are considered milder affective states. (3) Emotions are brief episodes that have a shorter duration than do moods and attitudes. The studies reviewed have not always explicitly adopted the criteria to differentiate emotions, moods, and attitudes. For instance, in some studies, states that are typically considered attitudes, such as ‘polite’, have been included as emotions (see Fig. 1). Given that emotions, moods, and attitudes are likely to produce different acoustic patterning (Scherer, 2003), we recommend that future research on emotional vocalizations distinguish emotional states from other affective states by using the three criteria outlined above.

### Methods for eliciting emotional vocalizations

#### Acted versus spontaneous expressions

The research included in our review has used actors who portray emotions, as well as spontaneous expressions from individuals reacting to a stimulus occurring in real time. Acted portrayals were mostly provided by speakers who were asked to vocalize a given carrier phrase (e.g., words, sentences) in a particular emotional state (e.g., Hammerschmidt & Jürgens, 2007; van Bezooijen, 1984). Speakers were often nonprofessionals (e.g., students), but were sometimes professional or amateur actors (see Table 1). Examples of spontaneous vocalizations include vocalizations produced during classroom discussions (Huttar, 1968) or radio interviews (Jürgens, Grass, Drolet, & Fischer, 2015).

**Fig. 5:**
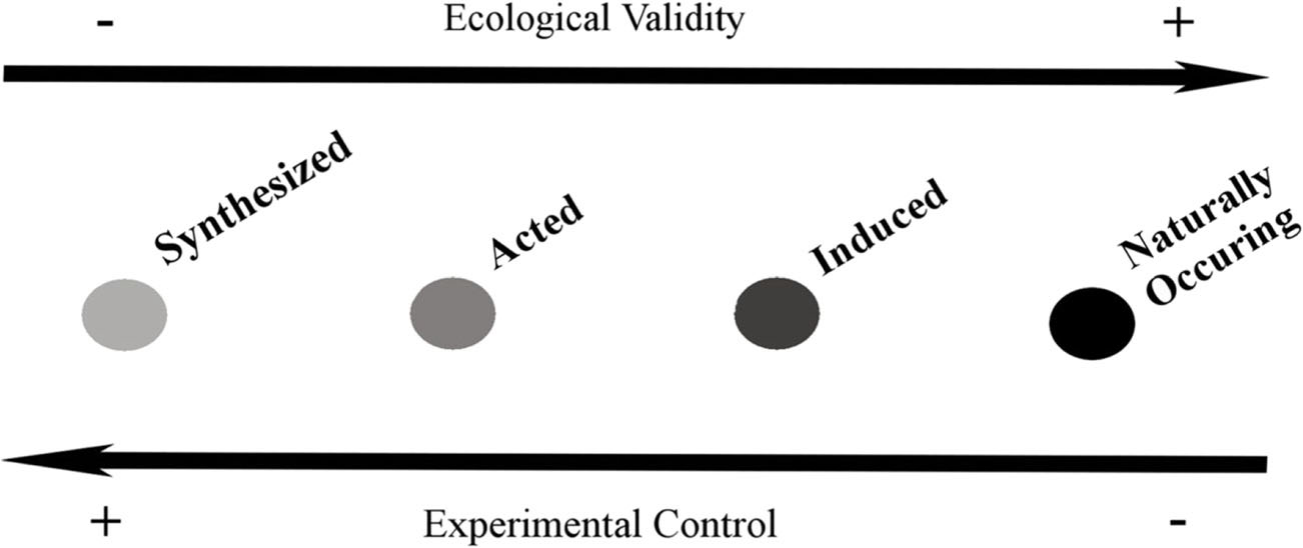
Comparison of different ways of eliciting emotional vocalizations in terms of experimental control and ecological validity

Compared with acted vocalizations, spontaneous emotional expressions are considered more natural and thus have higher ecological validity (e.g., Williams & Stevens, 1981). On the other hand, acted vocalizations provide more experimental control and allow for more accurate acoustic measures (e.g., Frank, Juslin, & Harrigan, 2005; see Fig. 5). In the context of the current review, an important question is whether acted and spontaneous expressions show different acoustic patterning for the same emotion. Previous research has compared acoustic properties of spontaneous and volitional laughter (Bryant & Aktipis, 2014; Lavan, Scott, & McGettigan, 2016; McGettigan et al., 2015; Neves, Cordeiro, Scott, Castro, & Lima, 2018; Wood, Martin, & Niedenthal, 2017) and has found that spontaneous laughter is higher in *pitch mean*, *maximum* and *minimum*. More generally, acoustic predictors of authenticity in nonverbal emotional vocalizations are higher and have more variable *pitch*, lower *harmonicity*, and less regular *temporal structure* (Anikin & Lima, 2017). Juslin, Laukka, and Bänziger (2017) compared acoustic features in acted and spontaneous emotional speech. Most of the features showed similar patterns, but subtle acoustic differences between acted and spontaneous happy speech were found in measures of frequency and temporal features (see also Banse & Scherer, 1996; Juslin & Laukka, 2003). Furthermore, their results pointed to intensity interacting with spontaneity in determining the acoustic features of vocal expressions of emotions. For instance, pitch variability was larger for acted than for spontaneous happy vocalizations in different intensity levels. These findings suggest that acted vocalizations are similar, but not identical, to spontaneous expressions. Thus, in future research, potential differences between acted and spontaneous vocalization, as well as the role of emotional intensity, should be considered (see also Sauter & Fischer, 2018).

#### Experimental induction of positive emotions

Another method for the production of emotional vocalizations is experimental induction of emotions in a laboratory setting. Researchers have elicited positive vocalizations by exposing participants to happy facial images (Barrett & Paus, 2002; Pell et al., 2015), computer games (Johnstone & Scherer, 1999), or music (Skinner, 1935). Although there are clear advantages to this experimental method, including the high degree of experimental control (see Fig. 5), it was the least commonly used method in the studies included in our review. Furthermore, this method was only used for the elicitation of happiness and joy.

Two major problems have been raised regarding emotion induction as a method of eliciting emotional expressions. First, emotion induction does not guarantee that speakers will experience or express the exact same emotion, because speakers’ reactions to a given induction method (e.g., using music) may vary with personal experience and personality (Scherer 1981). Second, it is challenging to induce strong emotions in laboratory settings (Laukka, 2004), which is important, given that the intensity of emotion influences the behavioural and physiological responses of the emotion thought to underlie changes in vocalizations (e.g., Brehm, 1999; Frijda, Ortony, Sonnemans, & Clore, 1992). Vocalizations of the same emotion at different levels of intensity have been shown to exhibit different acoustic features (see Juslin & Laukka, 2001). Thus, acoustic features associated with an emotion elicited by emotion induction might reflect acoustics of emotional vocalizations at low levels of intensity.

The study of vocal expression of positive emotions would benefit from capitalizing on empirically verified ways to induce high-intensity emotions in laboratory conditions, such as dyadic interaction tasks (e.g., romantic partners having conversations on enjoyable topics; Levenson, Carstensen, & Gottman, 1993), and virtual reality paradigms (e.g., Chirico, Ferrise, Cordella, & Gaggioli, 2018). Moreover, researchers could use self-report measures in combinations with physiological and behavioural measures to verify induction procedures, as well as to control for individual differences.

#### Synthesized/resynthesized positive emotions

The most highly controlled stimuli are the result of synthesized and resynthesized methods that systematically manipulate acoustic features (see Fig. 5). Synthesized speech is produced entirely by a computer, whereas resynthesized speech is generated from natural speech samples that are modified in terms of certain acoustic parameters. Acoustic features are related to happiness/joy (see Schröder, 2001, for a review), and tools have been created to resynthesize neutral voices with happiness/joy (e.g., Rachman et al., 2018). However, these recommendations are mostly limited to a single positive emotion category.

Synthesized/resynthesized vocalizations must first be modelled on human vocalizations that are elicited by one of the other methods. Synthesizing then allows for the manipulation of different acoustic features separately in vocalization samples. Once more acted and spontaneous samples of emotional vocalizations of different positive emotions are available, synthesizing and resynthesizing will offer powerful tools to examine the contributions of specific acoustic features.

#### Speakers

There is considerable variability in the sample sizes of the speakers whose emotional vocalizations have been analyzed in terms of acoustic characteristics. In our review, the number of speakers ranged from 1 to 63. Small sample sizes included spontaneous vocalizations obtained in natural situations (e.g., Huttar, 1968) or acted portrayals vocalized by professional actors (e.g., Breitenstein, Lancker, & Daum, 2001). The inclusion of only one or two speakers as emotion encoder could cause idiosyncratic effects (Laukka, 2004), rendering effects unreliable. Larger samples of speakers have consisted mostly of nonprofessional speakers (e.g., Costanzo, Markel, & Costanzo, 1969).

Studies have also varied in terms of the sex of the speakers, with some studies using only female encoders, others only male encoders, and yet others a combination of male and female encoders. Murray and Arnott (1993) emphasize that some pitch related speech parameters may depend on the sex of the speaker. For instance, pitch mean level is on average lower for male voices by about an octave, due to the difference in vocal fold length and thickness (Titze, 1994). When comparing females’ and males’ joyful vocalizations, females had higher and more variable *pitch* (Pollermand & Archinard, 2002). Furthermore, Szameitat et al. (2009) reported higher levels of *pitch* as well as higher mean frequencies of the first five *formants* in female than in male speakers during laughter.

Future research should include both male and female speakers with an adequate sample size to minimize the effects of sex and idiosyncratic variation. Restriction to one gender increases homogeneity, but limits generalizability. Furthermore, the inclusion of a large sample of speakers is important because articulatory factors such as laryngeal size and shape might cause interspeaker differences.

### Conclusions

Despite the importance of the human voice in communicating emotions, a systematic understanding of the acoustic features that convey information about positive emotions is lacking. In this review, we provide an overview of existing empirical research and offer a first attempt to integrate findings from this area of research. We first focused on comparisons between positive and neutral vocalizations. A happy voice is typically higher in pitch with higher *pitch variability* and *range*, *louder* with higher *loudness variability*, and higher in the first two *formant frequencies*. Variations in *pitch* show differences between high arousal emotions (joy) and low arousal emotions (tenderness and lust), when compared with neutral vocalizations. Second, we reviewed research comparing acoustic features across different positive emotions. Findings highlighted differences in *pitch*, *loudness*, and *speech rate*. The pattern of results for acoustic features fit the classification of positive emotions into emotion families: *Pitch* was high for epistemological emotions (amusement, interest, relief), moderate for savouring emotions (contentment and pleasure), and low for prosocial emotions (admiration). A similar pattern was found for *loudness* in speech prosody, but not in nonverbal vocalizations. Vocalizations of pride, and epistemological emotions (relief and interest) were produced at a faster rate than vocalizations of savouring emotions (pleasure and contentment) and a prosocial emotion (admiration). Some of these findings also map onto differences in levels of physiological arousal. For instance, *pitch* and *loudness* of high arousal emotions like joy and amusement were higher than low arousal emotions like pleasure and contentment. Similarly, joy and pride vocalizations were faster than pleasure and contentment. However, focusing merely on this broad dimension of arousal, fails to account for some of the systematic differences between distinct positive emotions.

Systematic comparisons of overlap and differences in acoustic features of vocal expressions of positive emotions can yield information about the key acoustic features characterizing positive emotions. It can also map out similarities and differences between different positive emotional states. The present results show that it is possible to differentiate specific positive emotions, as well as clusters of positive emotions, which may be characterized by different vocal signatures. Epistemological positive emotions are expressed with higher *pitch*, *loudness*, and *speech rate*. These source features are associated with how the respiration system generates and conducts the air flow. Our results suggest that when expressing epistemological emotions such as amusement and interest, we produce salient respiratory vocalizations. Such use of source features might serve the purpose of attracting others’ attention and function as salient social signals of emotional states. For instance, laughter with amusement might signal cooperative intent to others (e.g., Davila-Ross, Owren, & Zimmermann, 2009), and exclamations of interest might signal the motivation of wanting to learn more about something from a social partner (see Mortillaro, Mehu, & Scherer, 2011). In contrast, savouring positive emotions (contentment and pleasure) were lower in *pitch*, *loudness*, and *speech rate.* This might suggest that these emotions are perhaps not primarily linked to communicative functions, but rather serve adaptive functions for the person experiencing them.

We go beyond previous reviews (Juslin & Laukka, 2003; Murray & Arnott, 1993; Scherer, 2003) not only by reviewing a larger corpus of research (108 studies on vocal production of positive emotions) but also by thoroughly examining how that research was done—that is, examining the operationalizations of positive emotions as well as design features of this body of work. The systematic analysis of terminology, as well as the review of and recommendations for future research that we provided, are intended to help combat inconsistencies in the approaches employed in much of the research done to date. Considering the great variability in these features in the literature, we hope that our review will facilitate a more systematic approach to studying emotions in the voice in the future, and ultimately contribute to a better understanding of positive emotions.
